# Application of Species Distribution Models (SDMs) and Corridor Mapping for Conservation of an Endangered Charismatic Mammal: Brown Bear ( *Ursus arctos* ) in Iran

**DOI:** 10.1002/ece3.73590

**Published:** 2026-06-08

**Authors:** Farnoosh Kouchali, Bagher Nezami, Masoud Yousefi

**Affiliations:** ^1^ Department of Environment College of Environment Karaj Iran; ^2^ Research Group of Biodiversity and Biosafety Research Center for Environment and Sustainable Development Tehran Iran; ^3^ Faculty of Governance University of Tehran Tehran Iran; ^4^ School of Biology Damghan University Damghan Iran

**Keywords:** carnivore, conservation, habitat suitability, Iran, protected areas

## Abstract

The brown bear ( *Ursus arctos* ) is the largest terrestrial carnivore in Iran with a rapidly shrinking range. In this study, we assembled the most comprehensive and up‐to‐date distribution dataset of the brown bear in Iran. We applied Species Distribution Models (SDMs) and corridor mapping to identify this charismatic mammal's suitable habitats and its corridors across the Zagros and Alborz mountains to ensure this Endangered charismatic large carnivore in Iran. We also estimated the coverage of protected areas for the species' suitable habitats. Our data confirmed the presence of the species in 22 of 31 provinces of the country. The total suitable area for the species is 347,728 km^2^. We showed that significant parts of the suitable areas, 77%, of the brown bear's suitable habitats, are located outside of the protected areas network. Slope and vegetation were the most important variables shaping the species distribution, and dense forests in the absence of mountain areas are not suitable for the species. One practical result of this study is narrow, well‐defined corridors which are more feasible to validate in the field, monitor over time, and integrate into conservation planning. While the circuit method can provide valuable insights into corridor mapping, this method always produces wide corridors, which are challenging to prioritize for targeted conservation efforts for carnivores. Thus, we recommend linear corridors for carnivores' conservation planning.

## Abstract 2

We assembled the most comprehensive and up‐to‐date distribution dataset of brown bears in Iran. We applied Species Distribution Models (SDMs) and corridor mapping to identify this charismatic mammal's suitable habitats. We showed that 77% of the brown bear's suitable habitats are located outside of the protected areas network.

## 1 Introduction

Large carnivores, at the top of the food web, play a vital ecological role in ecosystem sustainability (Kuijper et al. 2016; Ripple et al. 2014; Tomita and Hiura 2024). However, all carnivores with varied sizes have low densities per unit area and are often threatened (Ripple et al. 2014). Hence, their conservation is widely emphasized as an effective protection strategy (Kunkel et al. 2013; Sergio et al. 2008). Strategies aimed at managing and protecting large carnivores are challenged due to their high biological needs, large home range, and extensive habitats (Ratti and Reese 1988), leading to their conflicts with humans (Chapron et al. 2014). Extensive planning and collaboration are needed to ensure that their habitat requirements are met (Rabinowitz and Zeller 2010). The protection of these species is an effective strategy against human development; since they are classified as an umbrella species and have a key role, it is possible to protect other species and the whole landscape in light of their extensive habitat needs (Chapron et al. 2014; Kunkel et al. 2013; Sergio et al. 2008; Simberloff et al. 1997).

Species Distribution Models (SDMs) have found important applications in ecology and conservation and were frequently used to identify suitable habitats as well as their habitat connectivity (Barbet‐Massin et al. 2012; Elith and Leathwick 2009; Guisan and Thuiller 2005; Guisan et al. 2017). These models and strategies help managers protect carnivores' source population, control core areas, and prevent their degradation (Poor et al. 2020; Mishra et al. 2022; Jones and Harris 2025). They also help managers plan conservation measures for sites with suitable habitat conditions but high mortality rates (Nielsen et al. 2006).

Brown bear ( *Ursus arctos* ), Eurasia's largest carnivore, has a large home range and occurs at low density in Iran (Marya Madadi et al. 2023). The species occurs in the rugged foothills of the Zagros, Alborz, and Caucasian mountains in the West, North, and Northwest of the country, respectively (Farahani and Asgharzadeh 2023). Brown bear population in Asia has suffered more damage than anywhere else in the world, due to numerous wars, habitat destruction due to more intensive development and severe land degradation, poaching, a severe reduction in prey (Servheen et al. 1998), and this species is classified as Endangered in Iran (Ashrafzadeh et al. 2023; Calvignac et al. 2009).

Over the last 50 years, the brown bear population and its habitats have experienced a significant degradation. This decline can be attributed to a multitude of factors, such as the aftermath of the Iran‐Iraq War, rapid human expansion, escalating population density, and extensive changes in land use throughout their natural range (Nezami and Farhadinia 2011). Consequently, given the species' once expansive but now swiftly diminishing and fragmenting breeding grounds, the brown bear, together with the gray wolf ( *Canis lupus* ), is now recognized as the most contentious carnivorous species in Iran (Farhadinia et al. 2019; Alireza Mohammadi and Kaboli 2016). The brown bear has one of the highest mortality rates of mammals in Iran due to poaching and habitat destruction (Madadi et al. 2020; Madadi et al. 2023). Although local studies have addressed this species over the past decade, little information on their distribution and habitat suitability across their entire dispersion area in Iran is available (Mohammadi et al. 2021).

Previous studies have built the species' habitat suitable models at different scales (Ashrafzadeh et al. 2018; Madadi et al. 2020; Mohammadi et al. 2021). While previous models are useful for the conservation of the brown bear, they are limited to a small part of the species distribution or they were built based on a small sample size (Almasieh et al. 2019; Ansari and Ghoddousi 2018; Ashrafzadeh et al. 2018; Ashrafzadeh et al. 2022). For instance, Almasieh et al. (2019) modeled habitat suitability and connectivity of the brown bear along the Iran‐Iraq border. In another study Ashrafzadeh et al. (2023) modeled distribution and identified determining factors for distribution of brown bear population in the Central Zagros Mountains. Besides, previously identified suitable patches and corridors are often large and continuous which are probably not in concordance with the patchy and fragmented habitat of the species in Iran. Many habitats along brown bear movement routes are likely to have high suitability; however, they may have low survival rates due to poor conservation and function as attraction sinks (Delibes‐Mateos et al. 2007; Naves et al. 2003) or ecological traps (Donovan and Thompson III 2001; Ratti and Reese 1988). Therefore, it is essential to identify this Endangered mammal suitable and priority habitats on the country scale and incorporate them in the conservation programs and management plans.

In this study, we collected and assembled the most comprehensive and up‐to‐date distribution database of brown bear distribution points across various ecoregions of Iran. We aimed to identify the species distribution on the country scale to be used to assess habitat suitability. Identifying habitat selection across diverse habitats, exploring their relationships with environmental integrity, and knowing the species distribution within the protected and non‐protected areas can help protect this large carnivore more effectively and ensure its long‐term survival (Crooks et al. 2011; Dickson et al. 2014; Santini et al. 2016).

## 2 Materials and Methods

### 2.1 Study Area

**FIGURE 1:**
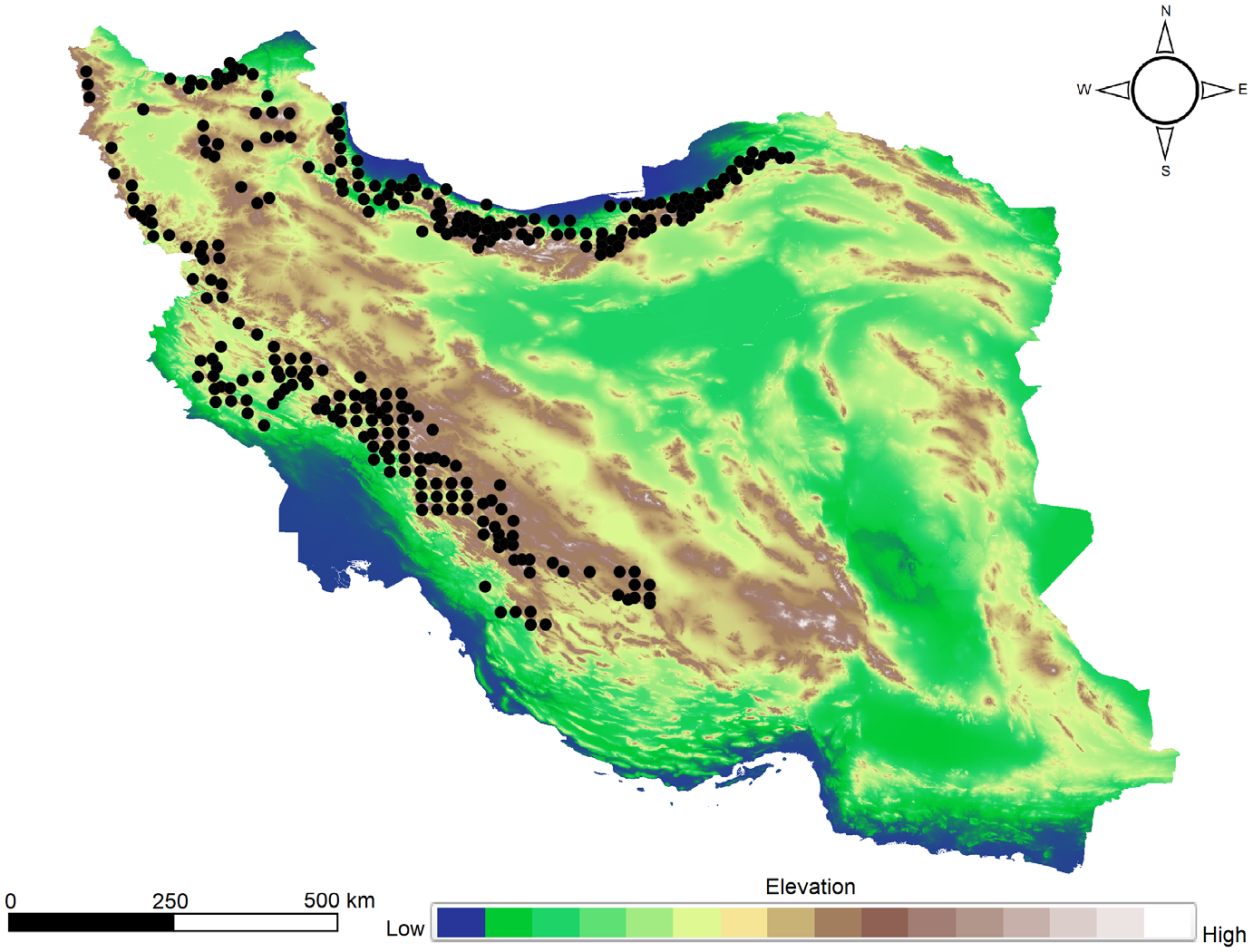
Study area along with presence points of the brown bear ( *Ursus arctos* ) in Iran. After applying the buffer on 800 presence points, 295 points remained.

This study was conducted across Iran, covering a surface area of 1,684,195 km^2^. Iran is located in southwestern Asia and is comprised of two large water sources in the north and south, two mountain ranges almost perpendicular to each other, and various environmental factors (Figure 1). Iran is a biodiversity‐rich country and hosts a high diversity of mammal species: 191 terrestrial mammals (Yousefi et al. 2023). Due to its diverse natural conditions, this country encompasses a unique diversity of mammals throughout the Middle East (Yousefi 2025).

The Iranian plateau has an arid climate, while the average annual temperature rises from the northwest to the southeast and varies from about 10°C in Azerbaijan to 25°C–30°C in Baluchistan in the south and southeast. The average annual rainfall in the country is 275 mm, varying from 2000 mm in Gilan Province to less than 100 mm in the central regions (Roohi 2013). Approximately 60% of Iran's land area is mountainous, primarily encompassing the Zagros and Alborz ranges, which host the highest biodiversity not only in the country but also in the entire Middle East (Shakoori et al. 2026). Therefore, the protected areas in these regions contain a higher diversity of species from different geographical ecoregions of the world, such as the brown bear, belonging to European fauna (Ziaei 2008). According to the latest official data published by the Department of Environment of Iran (DoE), the protected areas cover 177,571 km^2^, accounting for 10.77% of the total area of the country.

### 2.2 Data Collection

Distribution records of brown bear were collected by a combination of methods including direct observation of the team surveys, game guards, and DoE experts' observations which are as follows: the database in provincial DoE, direct observations of university experts and students, which are listed in the acknowledgments. In addition to direct observations, we collected the validated signs such as footprint. Distribution records were collected from 2014 to 2020 and the data consisted of 800 presence points (Figure 1). Comparing the collected distribution data with previously published distribution maps of the species showed that our data perfectly covered the entire distribution range of brown bear in Iran (Karami et al. 2012; Ziaei 2008).

To avoid the negative effects of autocorrelation, we thinned the distribution records of the species to 5 km (Sıkdokur et al. 2024). A total of 295 remaining points were used to model habitat suitability of the brown bear in Iran (Figure 1). Species distribution was analyzed after validation by field visits or carnivores' experts of each of 22 provincial offices of department of environment (Table 1).

**TABLE 1:** List of environmental predictors, abbreviations and VIF values.

No.	Variable	Abbreviation	VIF values
1	Annual temperature	(Bio1)	3.763
2	Annual precipitation	(Bio12)	3.544
3	Slope	Slope	2.516
4	Aspect	Aspect	1.126
5	Distance to roads	Dis_road	1.892
6	Distance to rivers	Dis_river	1.825
7	Distance to villages	Dis_village	1.966
8	Human Footprint Index	HFI	1.265
9	Normalized Difference Vegetation Index	NDVI	2.979

The Extent of Occurrence (EOO) of the brown bear in the country was calculated by the species presence data. EOO is defined as “the area contained within the shortest continuous imaginary boundary which can be drawn to encompass all the known, inferred or projected sites of present occurrence of a species, excluding cases of vagrancy. This measure may exclude discontinuities or disjunctions within the overall distributions of taxa. EOO can often be measured by a minimum convex polygon” (Boitani and Powell 2012). Finally, the collected data were categorized by province, type of region (free or protected), direct observation, number, and gender in Excel 2010. The outputs were prepared based on the points of presence using ArcMap 10.2 software.

### 2.3 Habitat Suitability Modeling

We used environmental variables related to climate, topography, vegetation, and human presence at 1 km spatial resolution which were identified to be important in shaping the species distribution in Iran (Table 1). For climate, we included mean annual temperature (Bio1) and mean annual precipitation (Bio12) which were obtained from Chelsa, at 30 arcsec, ~1 km spatial resolution (http://chelsa‐climate.org/). CHELSA is a climate bank data for the land surface areas which currently hosted by the Swiss Federal Institute for Forest, Snow and Landscape Research WSL (Karger et al. 2017). To consider topography, we added slope and aspect that are calculated from the Shuttle Radar Topography Mission (SRTM) digital elevation model at a 30 arcsec (~1 km) resolution (Jarvis et al. 2008).

To take into account landcover and human presence we used NDVI, distance to rivers, distance to roads, distance to villages, and human footprint index (Sıkdokur et al. 2024). Human footprint index is created by combining data on the extent of built environments, population density, electric infrastructure, crop lands, pasture lands, roads, railways, and navigable waterways and is freely accessible form the references presented here (Venter et al. 2016). Land use variables were obtained from Ghorbanian et al. (2020) land use map generated using GEE big data processing platform and a combination of Sentinel‐1 and Sentinel‐2 with 10 m spatial resolution (Ghorbanian et al. 2020). Village were extracted from a village dataset produced by Iran's government. A village is formed by at least 20 households. But for roads we considered primary and secondary roads which were obtained from OpenStreetMap (https://www.openstreetmap.org/). We also considered those roads connecting primary and secondary roads. Considering that the environmental predictors have different spatial resolution we aggregated and disaggregated them to 30 arcsec, ~1 km spatial resolution to which is the spatial resolution of climatic variables. We secured low multicollinearity (Quinn and Keough 2002) among the environmental predictors by calculating, a variance inflation factor (VIF < 10) using the “usdm” package.

The sdm package (Naimi and Araújo 2016) includes a set of models and options to evaluate model results and plan the potential distribution of the species in space and time. The data in this study were analyzed with the generalized linear model (GLM; (McCulloch and Nelder 1989)), boosted regression tree (BRT; (Friedman 2001)), multivariate adaptive regression model (MARS; (Friedman 1991)), flexible discriminant analysis model (FDA; (Hastie et al. 1994)), random forest model (RF; (Breiman 2001)), and maximum entropy model (MaxEnt; (Phillips et al. 2006)) in the sdm package (Naimi and Araújo 2016). Furthermore, an ensemble method was used to identify the habitat suitability by combining the results of the aforementioned models (Araújo 2007).

We generated 5000 pseudo‐absences by randomly sampling coordinates from the species' entire range. We calibrated the models using 80% of records drawn randomly and used as training data, and evaluated their performance using the remaining 20% of the data (test dataset). The area under the receiver operating characteristic (ROC) curve (AUC) was applied as a measure of the model performance (Giovanelli et al. 2010). AUC values range from 0 to 1; a value of 0.5 indicates that the performance of the model is not better than random, while values closer to 1.0 indicate better model performance.

### 2.4 Gap Analysis and High‐Priority Habitats

One of the challenges for conservation managers and conservation biologists is to assess the efficiency of protected areas in the conservation process (Rodrigues et al. 2004; Santini et al. 2014; Yusefi et al. 2016) and select new protected areas for more effective conservation of biodiversity (Jennings 2000; Rodrigues et al. 2004; Scott et al. 1993). Gap analysis describes how and to what extent the areas under management can cover the distribution of plant and animal species (Jennings 2000; Scott et al. 1993). A critical concern for decision‐makers is to distinguish between protection and development in the modern world (Yusefi et al. 2016). In this study, the result of the ensemble map from the previous section was used to assess the efficiency of the network of protected areas. To this end, we first classified the continuous habitat suitability map to a binary map using the 10‐percentile training presence logistic threshold which is frequently used in SDMs studies (Kafash et al. 2022; Liu et al. 2005; Sheykhi Ilanloo et al. 2021; Vale et al. 2014). Then, the classified map was overlaid with the layer of the map of the protected areas under the management of the DoE to determine the overlap between these areas and the habitats preferred by the brown bear (Kafash et al. 2022). The data from the observation of females with cubs were added to the final map to determine reproductive habitats. The source habitats were selected based on the observations of mothers and less than one‐year‐old cubs (Nezami 2008).

### 2.5 Habitat Connectivity Analysis

One of the strengths of the research is that identified suitable habitats were confirmed by the field surveys. Connection points and corridors were modeled based on the least‐cost pathway method (Kramer‐Schadt et al. 2011; Morato et al. 2014; Rabinowitz and Zeller 2010; Shams‐Esfandabad et al. 2021). The cost model prepared based on the inverse distance of suitability values was applied to the bear movements to identify priority corridors between protected areas. Then, the cost‐distance layer was used as the main input parameter for the modeling of the corridors. The least‐cost analysis was performed in QGIS (Shams‐Esfandabad et al. 2021). Here we used least‐cost pathway methods instead of circuit models because circuit models identify corridors which are often extremely broad (exceeding 70 km in width in some regions) and are challenging to prioritize for targeted conservation efforts. But as shown in our study least‐cost pathway methods can produce narrow, well‐defined linear corridors which are more actionable, more feasible to validate in the field, monitor over time, and integrate into conservation planning.

## 3 Results

### 3.1 Range and Distribution of Brown Bear in Iran

Our data confirmed the presence of brown bear in 22 of 31 provinces of the country (Table 2). The species had only seasonal vagrancy distribution in Yazd province in the centre of Iran. Accordingly, the distribution and observation of the bear populations in Iran belong to three distinct subpopulations: The Alborz, Zagros, and Caucasus (Arasbaran) mountains. The number of observations of the species in the Alborz subpopulation in the northern mountains was significantly higher than in the other two subpopulations. The Caucasian subpopulation contained a minimal distribution area of brown bear and was limited to the northwest. The Zagros subpopulation in the west of Iran had the most extensive distribution area compared to other subpopulations with the highest present points of the species in the unprotected areas. Brown bear occurrence was recorded in 61 protected areas, 10 national parks, 7 wildlife refuges, and 3 national monuments (Table S1).

**TABLE 2:** Unprotected habitats and associated counties where brown bear presence was recorded in Iran.

Province	Free areas (unprotected areas)
Zanjan	Tarom county (precinct Markazi and Chorzagh), Mahneshan county (precinct Markazi and Angoran)
West‐Azerbaijan	Piranshahr county (prescient Markazi and Lajan), Mahabad county (precinct Khalifan), Orumiye county (precinct Silvane and Soma‐e Baradost and Anzal), Chaldoran county (Markazi precinct), Khoy county (Safaeye precinct and Ghator precinct), Eshnavie county (Nalo's precinct), Sardasht county (Vazine precinct and Markazi precinct), Salmas county (Kohsar precinct)
Kohkiloye and Boyerahmad	Dena county (Patave precinct and Markazi precinct), Kohkiloye county (Dishmok precinct), Boyer Ahmad (Markazi precinct), Gachsaran county (Markazi precinct and Basht precinct)
Ardebil	Khalkhal county (Khoresh Rostam precinct and Markazi precinct and Shahrod precinct), Kosar precinct (Markazi precinct), Meshgin Shahr county (Meshgin‐e Sharghi precinct and Markazi precinct), Ardebil County (Markazi precinct), Germi county (Angot precinct), Pars Abad County (Aslan Doz precinct).
North‐ Khorasan	Mane va Samalqan county (Samalqan precinct and Markazi precinct)
Ilam	Dehloran county (Zarin Abad precinct), Dare shahr county (Badre precinct), Mehran County (Malekshahi precinct), Abdanan county (Sarab Bagh precinct), Ilam County (Markazi precinct and Chavar precinct), Shiravan and Chardavol county (Shiravan precinct)
East – Azerbaijan	Charoymaq county (Shadian precinct), Bostan Abad (Tikme Dash precinct and Markazi precinct), Shabestar county (Enzab precinct), Hashtrod county (Nazar Kahrizi precinct), Miane county (Kandovan precinct), Heris county (Markazi precinct), Tabriz County (Markazi precinct)
Chaharmahal and Bakhtiari	Ardal county (Markazi precinct and Miankoh), Shahr‐e kord county (Kiar), Kohrang county (Bazoft precinct and Markazi precinct), Lerdegan county (Markazi precinct and Meng precinct), Farsan county (Markazi precinct), Brojen county (Beldaji precinct).
Khozestan	Ize county (Markazi precinct and Dehdaz precinct), Masjed Soleyman county (Andika precinct), Dezfol county (Sardasht precinct), Lali county (Lali precinct), Bagh‐e Malek county (Seydon precinct)
Yazd	Khatam county (Markazi precinct)
Alborz	Savoj Bolaq county (Taleqan precinct), Karaj County (Asara precinct)
Lorestan	Kohdasht county (Darb Gonbad precinct, Markazi precinct and Romeshkan precinct), Khoram Abad county (Papi precinct, Dore chegeni precinct and Markazi precinct), Delfan county (Kakavand precinct and Markazi precinct), Pol ilani county (Mamolan precinct), Aligodarz county (Besharat precinct, Zezo mahro precinct and Markazi), Dorod county (Silakhor precinct), Azna county (Markazi precinct), Selse county (Markazi precinct), Nahavand county (Markazi precinct)
Kermanshah	Islam Abad‐e Gharb county (Markazi precinct), Pave County (Markazi precinct), Gilan‐e Gharb county (Govar precinct), Sahne county (Markazi precinct), Kangavar county (Markazi precinct), Kermanshah County (Mahidasht precinct)
Kordestan	Bane county (Namshir precinct and Neno precinct), Divan Dare County (Saral precinct and Markazi precinct), Marivan county (Sarshiv precinct and Markazi precinct), Sanandaj county (Kalatarzan precinct), Kamyaran county (Mochesh precinct), Saghez county (Sarshiv precinct), Sarv Abad County (Markazi precinct)
Fars	Ney riz county (Abade tashk precinct), Memsani county (Mahor ilani precinct), Khoram bid county (Mashhad marqab precinct), Sepidan county (Homayjan precinct and Markazi precinct), Boanat county (Sarchahan precinct and Markazi precinct), Shiraz county (Arjan precinct), Kazeron county (Kohmare precinct, Markazi precinct and jare and Baladeh precinct), Marvdasht county (Komfiroz precinct, Drodzan precinct, Seydan precinct and Sa'adat Abad precinct), Arsanjan county (Markazi precinct), Eghlid county (Hasan Abad precinct and Sade precinct), Estahban county (Markazi precinct)
Gilan	Rodbar county (Markazi precinct, Rahmat Abad and Bloukat precinct and Amarlo precinct), Rodsar county (Rahim Abad precinct), Siahkal county (Deylaman precinct and Markazi precinct), Amlash county (Markazi precinct), Tavalesh county (Asalom precinct, Markazi precinct and Havigh precinct), Masal county (Shanderman precinct), Astara county (Lavandovil precinct), Shaft county (Ahmad sar gorab precinct), Foman county (Sardar jungle precinct), Rodsar county (Rahim Abad precinct), Lahijan county (Otaghvar precinct), Rezvanshahr county (Pare sar precinct)
Semnan	Damghan county (Markazi precinct and Amir Abad precinct), Shahrod county (Bastam precinct), Semnan County (Mehdishahr precinct), Mayamei county
Mazandaran	Chalous county (Kelardasht precinct and Tonekabon precinct), Babol county (Banol precinct), Amol county (Dabodasht precinct, Larijan precinct and Markazi precinct), Nor county (Markazi precinct, Balade precinct and Chamestan precinct), Noshahr county (Kajor precinct), Sari county (Chardange precinct and Dodange precinct), Ramsar county (Markazi precinct), Savadkoh county (Markazi precinct and Shirgah precinct), Tonekabon county (Khoramabad precinct), Behshahr county (Yanesar precinct and Galogah precinct), Babol county (Bandpey‐e sharghi precinct)
Tehran	The mountain highest of the north
Golestan	Bandar‐e gaz county (Nokande precinct), AliAbad county (Kamalan precinct and Markazi precinct), Minodashht county (Galikesh precinct and Markazi precinct), Kordkoy county (Markazi precinct), Bandar‐e gaz county (Markazi precinct), Gorgan County (Markazi precinct and Baharan precinct), Ramian County (Markazi precinct), Azadshahr county (Markazi precinct and Cheshme saran), Kolale county (Markazi precinct)
Qazvin	Qazvin county (Tarom sofla precinct, Rodbar Shahrestan precinct and Rodbar alamout precinct)
Isfahan	Semirom county (Padena precinct and Markazi precinct), FereydonShahr county (Markazi precinct), Faridan county (Boein va miandasht precinct and Markazi precinct)

### 3.2 Habitat Suitability Models

**FIGURE 2:**
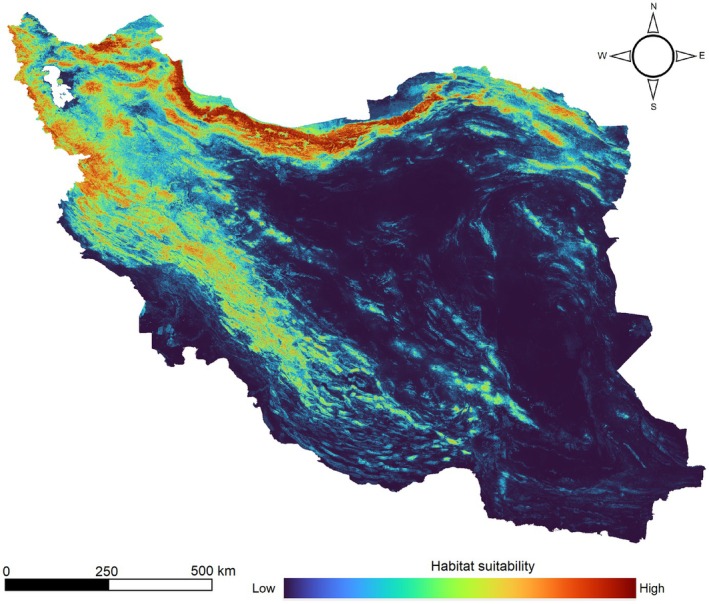
Habitat suitability map of the brown bear ( *Ursus arctos* ) in Iran. The model is produced by ensemble of following five models: MaxEnt, Mars, BRT, FDA, and GLM models.

The results showed that all models (GLM, BRT, MARS, and FDA) performed well according to the AUC metric (Table 3). We found that Alborz, Zagros, and Kopet Dagh mountains have high suitability for the brown bear in Iran. Among them, Alborz mountainous was identified as the region with highest suitability (Figure 2).

**TABLE 3:** AUC values in the different models.

Model	AUC value
RF	0.92
MaxEnt	0.91
MARS	0.91
BRT	0.9
FDA	0.9
GLM	0.9

### 3.3 Variable Importance

Vegetation and slope variables had the highest relative importance in the habitat suitability model of the species in Iran. The slope variable is the most significant variable concerning the presence and suitability of the species' habitat. The whole identified suitable habitats were mountainous. Thus, dense historic plain forests in the north of the country, the southern of the Caspian Sea and the northern slopes of Alborz Mountain, are not suitable for the species.

### 3.4 Protected Areas Coverage

**FIGURE 3:**
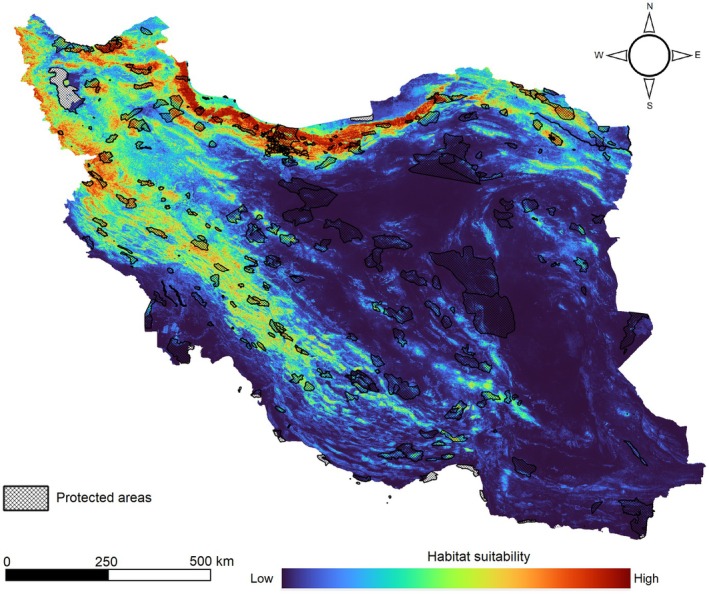
Protected areas coverage for the suitable habitat of brown bear ( *Ursus arctos* ) in Iran.

The cut of value for the suitable/unsuitable habitat was 0.26 based on the 10‐percentile training presence logistic threshold. The separation level between suitable and unsuitable habitats was determined. Accordingly, 23.38% of the area of the country (347,728 km^2^) was suitable for the species. Overlaying the network of protected areas with the habitat suitability map indicated that 34.53%, 18.27%, and 6.32% of suitable habitats were covered by protected areas, national parks, and wildlife refuges, respectively. The overlap of suitable habitats with the protected areas network in the species' extent of occurrence indicated significant parts of the suitable areas are outside of the protected areas network (Table 4 and Figure 3).

**TABLE 4:** The overlap of suitable habitats and protected areas network.

Protected area	Number	Area (Ha)	Area of suitability habitat	Suitability habitat (%)
National Park	29	2,001,629	365,697.62	18.27
Wildlife Refuge	44	5,595,749	353,651.34	6.32
Protected area	168	9,473,070	3,271,051.07	34.53
Total	241	17,070,448	3,990,400.03	23.38

### 3.5 Corridors and High‐Priority Habitats

**FIGURE 4:**
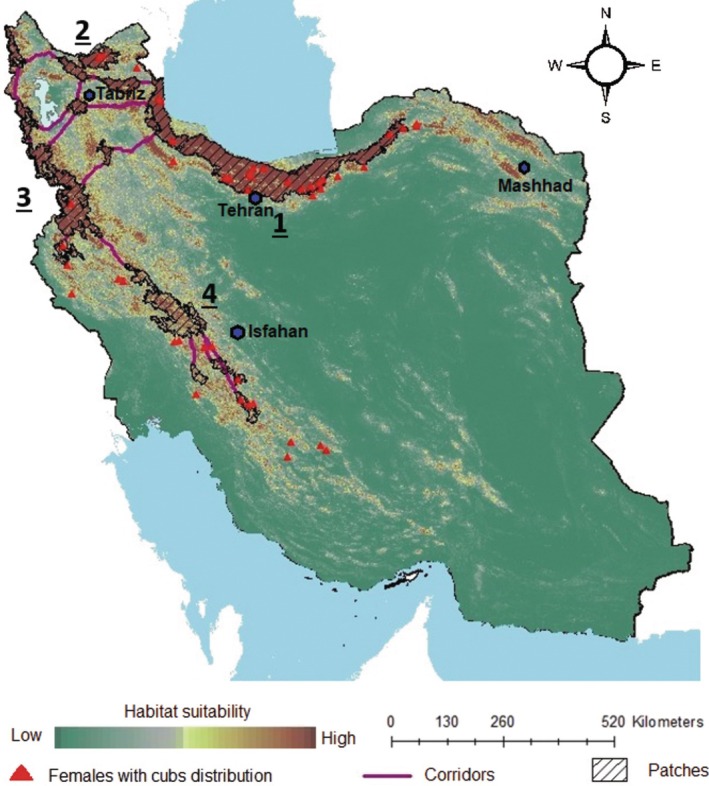
Corridors and source patches of brown bear ( *Ursus arctos* ) in Iran.

The brown bear's largest and most integrated habitat area (Figure 4), patch number (1) is located in the Alborz Mountains. It extends from the easternmost part of Alborz in Golestan province to the western part at the junction with the Caucasus ecoregion. The four core habitats, identified with high to medium suitability, are (Figure 4): (1) Alborz ecoregion, including three provinces of Golestan, Gilan, and Mazandaran. (2) East‐Azerbaijan and Ardabil provinces ecoregion. (3) West of Kurdistan, West‐Azerbaijan, and northwest of Kermanshah provinces ecoregion. (4) Lorestan and north of Chaharmahal and Bakhtiari provinces ecoregion. The data showed that 93.2% of the Alborz mountains in the north are composed of suitable habitats, accounting for 29% of the total suitable habitats of the country. However, only 6.4% of this area was protected.

Mothers and cubs were observed in 15 areas, including 10 protected areas, 1 wildlife refuge, and 4 national parks. Most observations of mothers with cubs were reported from the Central Alborz Protected Area, and Golestan National Park, in the central and eastern parts of the Alborz ecoregion, respectively. There were also 19 areas where few mothers' and cubs' observations were reported, but they are still unprotected (Table 2 and Table S1).

Analysis of habitat connectivity showed that the Alborz subpopulation had the highest connectivity to other subpopulations regarding the suitability and integration of habitats. The three subpopulations identified in Iran, Alborz, Zagros, and Caucasia, are linked through these connections along with the mountainous areas in the northwest. Field surveys indicated that the majority of the movements between subpopulation regions occurred in spring and especially in summer. In terms of the range of suitable areas, the number of reproductions, and the length of corridors, the Alborz subpopulation was shown as the core area for the brown bear in Iran, and protected areas in this ecoregion indicated to play a key role as the source habitats.

## 4 Discussion

### 4.1 Distribution and Habitat Suitability of the Brown Bear in Iran

In this study we used the most comprehensive and up‐to‐date distribution dataset of the brown bear in Iran to develop habitat suitability and corridor maps of the species to inform conservation decisions. Analyses of the data showed that despite the suitability of extensive mountainous areas along the north, northwest, and west of the country, only a small portion of these habitats is under protection. According to the results, the most suitable habitats for the brown bear are areas with high topographic unevenness, dense vegetation, and remoteness from human settlements (Mohammadi et al. 2021). Although the modeling data confirmed the existence of some large and well‐integrated areas, especially along with the Alborz Mountains, field surveys indicated that they are highly fragmented at the local level due to the expansion of man‐made structures in the areas (Sharifi et al. 2023). Therefore, there are high casualties, especially in late summer and early autumn, when the species is in hyperphagia and the species' dispersion and home range increases (Farhadinia et al. 2010).

Our study revealed that vegetation and slope are the primary predictors influencing the distribution of the species, both exhibiting positive correlations, which is in line with previous studies (Mohammadi et al. 2021). The species demonstrates a preference for steeply sloped terrains, as these areas offer not only shelter but also ideal sites for dens (Goldstein et al. 2010; Sharifi et al. 2023). Conversely, flat terrains or regions with gentle slopes tend to be more conducive to human development, with the majority of such activities occurring in these less inclined areas, particularly in the north of Iran (Asgharzadeh et al. 2023; Farahani and Asgharzadeh 2023). Brown bears strongly rely on rugged and mountainous habitats, deliberately avoiding flat and level areas, regardless of other favorable conditions (Goldstein et al. 2010). Most protected areas in the north and west of Iran, along with the Alborz, Zagros, and Caucasian mountains, are mountainous areas with complex topographic conditions. Thus, protected areas have more density and higher species richness in these parts of the country (Darvishsefat 2006; Maria Madadi et al. 2021), supporting more brown bear populations (Farhadinia et al. 2010).

The brown bear populations in all three northern, north‐western, and western ecoregions are severely affected by humans. These conflicts, especially in the western population in the Zagros Mountains and northwest in the Caucasus, are associated with more severe pressure due to more land‐use changes, less dense vegetation, lower habitat suitability, lower numbers of game guards, and lower protection facilities (Soofi et al. 2022). The sharp decline and low population density of the brown bear, even in protected areas, have been evident in recent decades. The dispersion of villages, whose entire lives depend on natural resources for agriculture, animal husbandry, horticulture, beekeeping, and firewood harvesting, is immense (Maria Madadi et al. 2021). There are a greater number of densely distributed villages and human populations in the Alborz Mountains in the north compared to the north‐western and western areas.

### 4.2 Protected Areas Coverage

Previous studies that evaluated protected area network efficiency indicated that existing protected areas are not effective enough to ensure long‐term conservation of biodiversity in Iran (Ahmadi et al. 2020; Kafash et al. 2022). Here we estimated protected area coverage for the suitable habitat of the brown bear and showed that most of the suitable habitat of the species is located outside of protected areas, particularly in the Alborz Mountains. Thus the protected areas of Iran may not be effective enough to conserve a viable population of brown bear (Mohammadi et al. 2021). In addition, these areas are not efficient in protecting other large carnivores in Iran (Ahmadi et al. 2020). Accordingly, although the network size of protected areas is increasing, the high mortality rate of brown bear and other carnivores outside protected areas (Madadi et al. 2023) indicates that these areas do not have enough efficiency to protect large carnivores (Visconti et al. 2019). The most common cause of habitat fragmentation in Iran is the road network (Mohammadi and Kaboli 2016), which facilitates access to all areas, resulting in the loss of brown bear due to poaching and road accidents.

Protected areas and national parks can play a vital role in protecting the brown bear populations in Iran. The density of the human population and far more land use at local levels have prevented the movement of subpopulations between protected areas in the country. In all three subpopulations, the absence of safe corridors between habitats causes limited movement and inefficient protection of this species (Sharifi et al. 2023). The results of connectivity analysis are consistent with genetic studies on Iranian bears (Ashrafzadeh et al. 2016; Yousefi et al. 2023). As they are genetically distinct populations but they are not completely isolated. The sharp decline of brown bear in Zagros and Caucasus populations, even in protected areas, indicates a high conflict with local people and insufficient protection due to the low number of rangers and weak protective equipment. Therefore, defining new protected areas, especially along the identified corridors, acting as stepping stones, can contribute to species protection (A. Mohammadi et al. 2021). It should be noted that some corridors might cross unsuitable habitats but we suggest that future field surveys can monitor these corridors.

### 4.3 Source Habitat Connectivity

Due to the higher frequency and high reproduction of the brown bear in Golestan National Park and Central Alborz Protected Area (Patch 1 in Figure 4) (Gutleb and Ziaie 1999; Kiabi et al. 1994; Nezami Balouchi 2014), the Alborz Mountains are home to the main population of brown bear (Blanford 1876; Gutleb and Ziaie 1999; Lay 1967; Nezami 2008; Nezami and Farhadinia 2011) and are considered to be the core area for the brown bear in Iran (Gutleb and Ziaie 1999; Nezami and Farhadinia 2011). Thus, the brown bear population survival in Iran depends on protecting the Alborz mountainous areas in the north of the country (Kouchali et al. 2018). According to the results, several corridors connect the three subpopulations, with the highest connectivity running from Alborz to the other populations (Table 2).

Carnivores are facing important challenges for survival outside protected areas in Iran (Madadi et al. 2023). Thus, it should be noted that conserving carnivores is essential not only within protected areas but also in human‐dominated landscapes because protected areas alone may not provide enough space for carnivores to have viable populations (Madadi et al. 2023). We identified several free zones in 19 counties where some mother‐cub observations have been reported while they are still unprotected. Unfortunately, brown bears are being killed in these areas. We believe that this is the most important finding of this study; thus, an urgent strategy is necessary to ensure the long‐term survival of the brown bear in Iran. One particular solution is to turn the unprotected habitats into seasonal protected areas and place strict conservation during the breeding season.

Patch 1 (Figure 4) shows an area important to serve as a stepping stone, the protection of which plays a vital role in the distribution of populations and genetic diversity of the brown bear in Iran. Brown bears have great potential for extensive distribution (Ambarli and Bilgin 2008; Nezami et al. 2018). However, even if covered with forests, flat and plain areas are not suitable for habitat connectivity (Mohammadi et al. 2021). These areas are often affected by local communities, human activities, and other related threats (Ashrafzadeh et al. 2018) and have a fragmented situation at the local level. Some protected areas in the southern and western parts of the Alborz mountains exist, and there is a high connectivity potential between the three subpopulations. Due to the high dispersal ability of brown bear, especially males (Ambarli and Bilgin 2008), three populations can be expected to be connected through these stepping stones.

## 5 Conclusion

In this study, we used the most comprehensive and up‐to‐date distribution dataset of the brown bear in Iran and identified the species' priority habitats and corridors of this Endangered charismatic large carnivore in Iran. Results of this study can significantly contribute to the conservation of the brown bear in Iran. We believe that this dataset alone is a valuable contribution to the current understanding of the global distribution of the brown bear and it has the potential to update the IUCN red list map. Conservation of suitable and priority habitats particularly free source areas identified in this study have high management priority and will play a critical role in the long‐term survival of the species. To reduce the effect of the fragmentation of habitats and the separation of population patches, it is necessary to protect corridors with the definition of new protected areas and increase the level of awareness of local communities (Yousefi 2025). An effective strategy for protecting large carnivores such as the brown bear with long seasonal movements, low density, high conflict, and extensive conservation requirements would be to connect the habitat and define new protected areas or community conserved areas along with the animal movement path.

One practical result of this study is narrow, well‐defined corridors which are more feasible to validate in the field, monitor over time, and integrate into conservation planning. While circuit method can provide valuable insights to corridor mapping, this method always produces wide corridors which are challenging to prioritize for targeted conservation efforts for carnivores. Thus, we recommend linear corridors for carnivores' conservation planning.

## Author Contributions

**Farnoosh Kouchali:** data curation (equal), formal analysis (equal), methodology (equal), resources (equal), software (equal), writing – original draft (equal). **Bagher Nezami:** data curation (equal), funding acquisition (equal), investigation (equal), methodology (equal), project administration (equal), resources (equal), supervision (equal), validation (equal), writing – original draft (equal), writing – review and editing (equal). **Masoud Yousefi:** methodology (equal), validation (equal), visualization (equal), writing – review and editing (equal).

## Funding

This study was supported by Iran National Science Foundation (INSF): 4003852.

## Conflicts of Interest

The authors declare no conflicts of interest.

## Supporting information


**Table S1:** Distribution of Brown bear in source and sink habitat.

## Data Availability

The manuscript file contains all data and researchers can contact the corresponding author (Bagher Nezami: nezamibagher@gmail.com) on reasonable request. Data can be accessed via this link https://doi.org/10.5061/dryad.k98sf7mnd in the Dryad repository of research data.
