# Supplementary material for: Application of Species Distribution Models (SDMs) and Corridor Mapping for Conservation of an Endangered Charismatic Mammal: Brown Bear ( Ursus arctos ) in Iran
Source: Ecol Evol. 2026 Jun 8;16(6):e73590. doi: 10.1002/ece3.73590 (PMC13244082; doi:10.1002/ece3.73590)
Supplement: Supplementary file 1 — Table S1: Distribution of Brown bear in source and sink habitat. [file ECE3-16-e73590-s001.docx]

Supplementary file for:

**Application of Species Distribution Models (SDMs) and corridor mapping for conservation of an Endangered charismatic mammal: brown bear (*Ursus arctos*) in Iran**

Farnoosh Kouchali, Bagher Nezami, Masoud Yousefi

**Table S1. Distribution of Brown bear in source and sink habitat**

| Protected Area | Name |
| --- | --- |
| National Park | Source area: Bamo, Dena, Golestan Sink Habitat: Kental, Lar, Salouk, Arasbaran, Paband, Kiasar, the boundery of Orumie N.P. |
| Protected area | Sorce habitat: Arasbaran, Shimbar, Alborz markazi, Lisar, GHorkhod, Sefidkoh, Parvar, ghalaje, gheysari, Gasht-e rodkhan va Siaah mazegan.  Sink habitat: Bozin merkhil, Dena, Haraz, Zave-e yek, Zav-e Do, Love, Vaz, Abshar-e Margon, Jangal-e Abr, Oshtorankoh, Jahannama, Helen, Angoran, Sorkh abad, Mirabad, Marakan, Dena-e Sharghi, Koh-e Khamin, Koh-e Dil, Siok, Aghdagh, Kabirkoh, Dinarkoh, Kaghazkanan, Dizmar, Sahand, Sabz koh, Shalo va Mongasht, Bagh Shadi, Male gale, Siahrod, Chahar bagh, Arjan va Parishan, Koh-e Khiz va Sorkh, Bisoton, Manasht va ghalarang, Beleskoh, Asas, Shesh rodbar, Bola, Hezar jarib, Serolat va Javaherdasht, Kheybus va Anjilsee, Abshar-e shirgah, Tang bostanak, Varjin, Bozghosh, Yari ghari, Kolak, Abdorazagh, Kohsalan. |
| Wildlife Refuge | Source Habitat: Bakhtegan, Dodange  Sink Habitat: Kiamaki, Bisoton, Khosh yeylagh, Angoran, Broeie, Zarivar. |
| National Monument | Alam koh, Ghole Damavand, Ghole Sabalan. |
| Free Zone | Source Habitat: Tarom Sofla No hunting area, 7 km far of Salok protected area, 7 km far of Roshankoh No hunting area, Padena-e Semirom No hunting area, In the bundery of Alborz Markazi protected area, 2 km far of Kiasar national park, 1 km far of Bola protected area, 4 km far of Dena protected area, 15 km far of Parvar protected area, 11 km far of Qeisari protected area, 2km far of Taal No hunting area, between Zav protected area and Neyshak no hunting area, 11 km far of Kosalan protected area, 2km far of Tal ova Shirband no hunting area and Sefidkoh no hunting area, Near the Shimbar protected area, Near the Alamot No hunting area, Vanak no hunting area, Near the Lefor no hunting area and Haraz protected area. |
